# Correction: Effect of temperature and moisture contents on dielectric properties at 2.45 GHz of fruit and vegetable processing by-products

**DOI:** 10.1039/d0ra90057e

**Published:** 2020-05-19

**Authors:** Katalin Solyom, Pilar Rosales López, Patricia Esquivel, Ana Lucía Vásquez-Caicedo

**Affiliations:** Fraunhofer Institute for Interfacial Engineering and Biotechnology IGB Nobelsrtaße 12 70569 Stuttgart Germany solyomkatalin@gmail.com; School of Food Technology, University of Costa Rica 2060 San Pedro Costa Rica

## Abstract

Correction for ‘Effect of temperature and moisture contents on dielectric properties at 2.45 GHz of fruit and vegetable processing by-products’ by Katalin Solyom *et al.*, *RSC Adv.*, 2020, **10**, 16783–16790, DOI: 10.1039/C9RA10639A.

The authors regret that the name of one of the authors (Ana Lucia Vasquez-Caicedo) was shown incorrectly in the original article. The corrected author list is as shown above.

The Royal Society of Chemistry apologises for these errors and any consequent inconvenience to authors and readers.

## Supplementary Material

